# A comprehensive study of olefin metathesis catalyzed by Ru-based catalysts

**DOI:** 10.3762/bjoc.11.192

**Published:** 2015-09-29

**Authors:** Albert Poater, Luigi Cavallo

**Affiliations:** 1 Institut de Química Computacional i Catàlisi and Departament de Química, Universitat de Girona, Campus Montilivi, 17071 Girona, Catalonia, Spain; 2 KAUST Catalysis Center, Physical Sciences and Engineering Division, King Abdullah University of Science and Technology, Thuwal 23955-6900, Saudi Arabia

**Keywords:** cis, density functional theory (DFT), N-heterocyclic carbene, olefin metathesis, ruthenium

## Abstract

During a Ru-catalyzed reaction of an olefin with an alkylidene moiety that leads to a metallacycle intermediate, the cis insertion of the olefin can occur from two different directions, namely side and bottom with respect to the phosphine or N-heterocyclic ligand (NHC), depending on the first or second generation Grubbs catalyst. Here, DFT calculations unravel to which extent the bottom coordination of olefins with respect is favored over the side coordination through screening a wide range of catalysts, including first and second generation Grubbs catalysts as well as the subsequent Hoveyda derivatives. The equilibrium between bottom and side coordination is influenced by sterics, electronics, and polarity of the solvent. The side attack is favored for sterically less demanding NHC and/or alkylidene ligands. Moreover the generation of a 14-electron species is also discussed, with either pyridine or phosphine ligands to dissociate.

## Introduction

Organic synthesis is based on reactions that drive the formation of carbon–carbon bonds [1]. Olefin metathesis represents a metal-catalyzed redistribution of carbon–carbon double bonds [2–6] and provides a route to unsaturated molecules that are often challenging or impossible to prepare by any other means. Furthermore, the area of ruthenium-catalyzed olefin metathesis reactions is an outstanding field for the synthesis of C–C double bonds [7–9]. After the discovery of well-defined Ru-based (pre)catalysts, such as (PCy_3_)_2_Cl_2_Ru=CHPh [10], first by Grubbs and co-workers the range of these catalysts was broadened because of their tolerancy towards heteroatom ligands and the possibility to work under mild conditions.

**Scheme 1:**
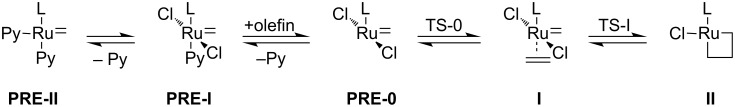
Mechanism of the olefin metathesis.

The next step was the substitution of one phosphine group by an N-heterocyclic carbene, NHC, which strongly increases the activity [11–15]. And, furthermore, a detailed comprehensive analysis of the chemical mechanics of these Grubbs catalysts was required. Once a better understanding of the performance of such catalysts was achieved, a rational design of new more active catalysts was envisaged [16–19]. Despite experimental [20–24] and theoretical [8–9,25–26] insights during the last two decades, demonstrating the mechanism in Scheme 1, there are still missing parts in the understanding. Anyway, it is also confirmed that the first steps go through a dissociative mechanism instead of an associative, i.e., the entering olefin arrives after the extraction of two pyridine groups. Most of studies are related to phosphine groups instead of pyridine groups, but the function of both chemical groups is the same.

**Scheme 2:**
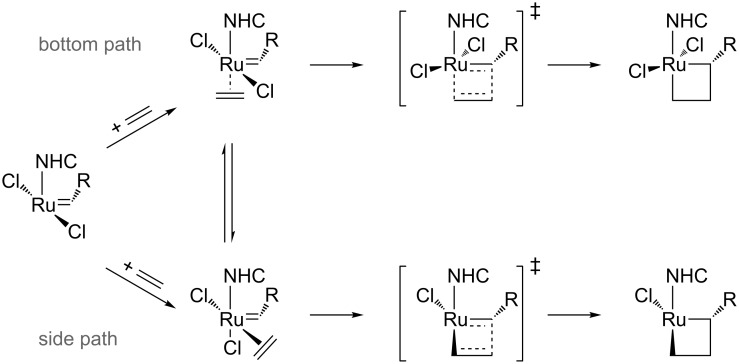
Possible side or bottom mechanism of the insertion of the olefin.

The release of a pyridine or phosphine group generates a 14-electron (14e) species, which binds to an olefin, coordinated cis to the alkylidene. The exchange of the leaving group by an olefin is found to be mainly dissociative [4,27–29], towards the associative or the concerted mechanisms [30]. The next metallacycle intermediate is due to the reaction of the olefin with the alkylidene moiety. Nevertheless in the cis insertion of the olefin, this olefin can enter from two different directions, side and bottom, displayed in Scheme 2. Regarding this cis insertion of the olefin, even though most papers favor the side insertion of the olefin (see Scheme 2) over the bottom insertion [31], both pathways might exist depending on the ligands and type of olefin. Since the year 2000 many papers try to unravel the preference for the bottom attack and how to favor the cis one [8,32–34], with an open debate still for the(NHC)Ru-based catalysts [35–38]. The postulated binding of the substrate can be preferentially trans to the NHC ligand (bottom path in Scheme 2) or cis to this ligand with the simultaneous shift of an halogen group to a trans position (side path in Scheme 2).

**Scheme 3:**
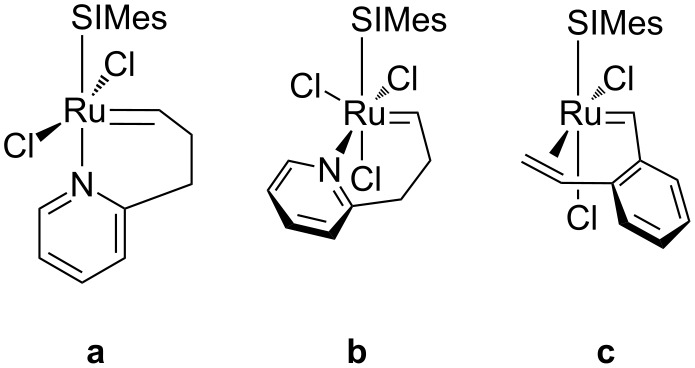
Ruthenium catalysts, bottom-bound (**a**) or side-bound (**b** and **c**).

Bearing the general acceptance [39–45] that olefin metathesis with Ru-catalysts starts from a bottom-bound olefin complex because of energetics, i.e., reporting higher energies for the possible side-bound olefin complexes, Piers and co-workers demonstrated the bottom-bound geometry for a Ru-cyclobutane model compound by NMR data [46]. However, Grubbs and co-workers supported the side-bound pathway [47]. And this sort of discrepancy is displayed in Scheme 3, where the same catalyst shows two conformations, **a** and **b**, bottom and side, respectively. Next, by means of DFT calculations Goddard and co-workers indicated clearly that solvent effects were of paramount relevance to the relatively high stability of **b** [48], while in the gas phase structure **a** was much favored [13]. Moreover, Grubbs and co-workers reported the X-ray structure of the model compound **c**, which clearly indicates that the olefin is side-bound to Ru [49].

**Scheme 4:**
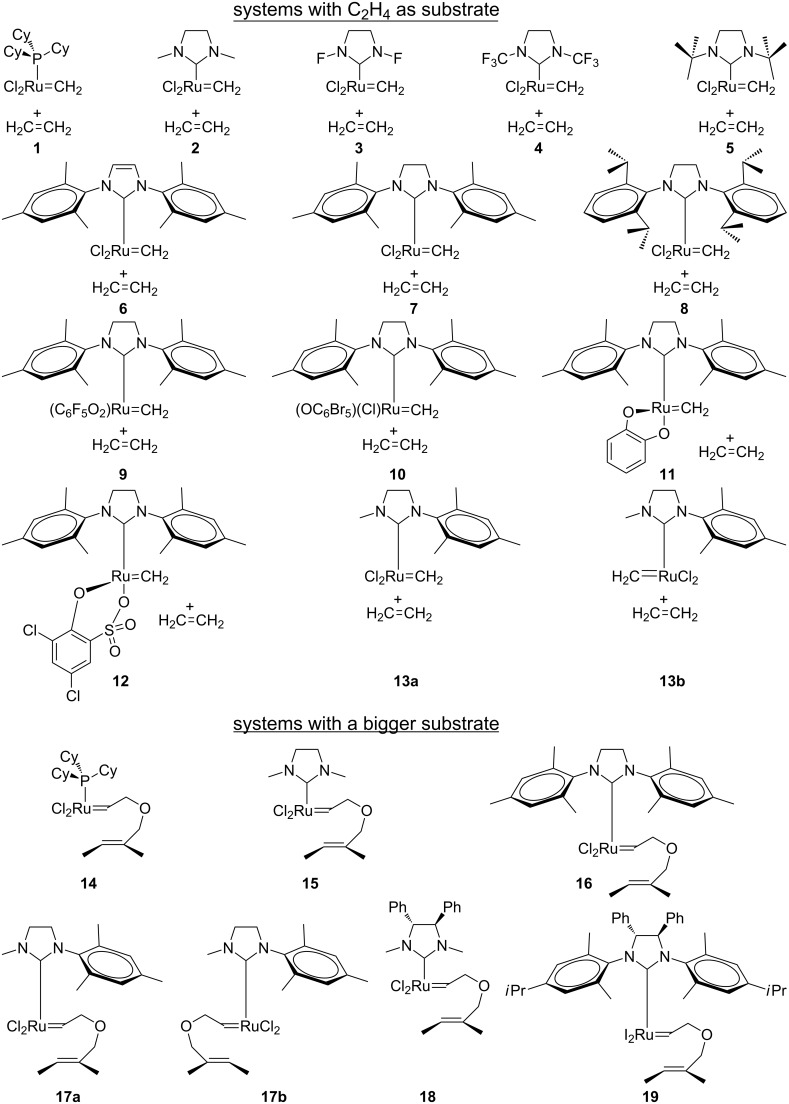
Studied systems.

Next, Correa and Cavallo discussed about the feasibility of the side conformation for the classical olefin metathesis catalysts **7**, **16**, and **19** displayed in Scheme 4 [8], concluding that the bottom/side equilibrium is based on a delicate balance between electronic, steric, and solvent effects. Particularly sterically demanding substituents of the NHC and bulky olefins clearly favor the side reaction pathway. Moreover this study corroborated the validity of BP86 for these second generation Grubbs catalysts and the conclusion was that any generalization could be done about the side/bottom stability of the coordination intermediate, as well as it is not possible for the first Grubbs catalysts [9]. Overall, the inclusion of a polar solvent and the absence of strong steric effects, i.e., with less bulky ligands (less than SIMes) and/or substrates [50–51], favored the side-bound structures over the bottom-bound ones as suggested by Goddard and Grubbs, respectively [16–18]. This is a possible explanation of why there is experimental evidence for some structures with side confirmation. On the other hand, complex **19** is an example of an asymmetric catalyst, suggesting that the NHC ligand is the source of asymmetry [52].

In this paper we contribute in the understanding of the side- and bottom-bound coordination intermediates and the stability of the corresponding transition states as well as the relative stability of the next metallacycle formed for (NHC)Ru(X)_2_ catalysts through a DFT approach with the BP86 functional. The comparison with the first generation Grubbs catalysts with a phosphine group instead of an NHC and the game based on the possible electronic and steric possibilities of the NHC will center our interests. All studied systems are displayed in Scheme 4. System **7** has been thoroughly studied because of its simplicity, playing especially with the NHC ligand, replacing one or both mesityl groups by CH_3_, CF_3_, *t*-Bu, H, F, and combinations between them to also observe the effects of an asymmetric NHC ligand. Furthermore this system **7** was also taken to discuss about the evolution of the precatalysts, **PRE-II**, **PRE-I** and **PRE-0** (see Scheme 1). This can corroborate the dissociative mechanism of the entering olefin, a step which has been taken into account. Then complex **15** reveals a more representative substrate, and is useful to focus on the study of steric effects of the substrate, reinforced the steric hindrance aspect replacing one or both mesityl groups of NHC ligand by methyl groups. Bearing the asymmetric catalysts **17**–**19**, particularly complex **19** can be compared to **16** but introducing asymmetry [17,53]. Then complexes **1** and **14** are representative of the first generation Grubbs catalysts. Finally, several systems are displayed to get insight into the typical properties of free halogen catalysts [54–55].

## Computational details

The density functional calculations were performed on all the systems at the GGA level with the Gaussian03 set of programs [56]. Two popular functionals, B3LYP and BP86, were considered. B3LYP calculations utilize Becke's three parameter hybrid exchange functional together with the correlation functional of Lee, Yang and Parr [57–59]. For BP86 calculations, gradient corrections were taken from the work of Becke and Perdew [60–62]. The electronic configuration of the molecular systems was described by the standard SVP basis set, i.e., the split-valence basis set with polarization functions of Ahlrichs and co-worker, for H, C, N, P, O, S, F, Cl and Br [63]. For Ru and I we used the small-core, quasi-relativistic Stuttgart/Dresden effective core potential (standard SDD basis set in Gaussian03) basis set, with an associated (8s7p6d)/[6s5p3d] valence basis set contracted according to a (311111/22111/411) scheme [64–66].

The geometry optimizations were performed without symmetry constraints, and the nature of the extrema was checked by analytical frequency calculations. Furthermore, all extrema were confirmed by calculation of the intrinsic reaction paths. The energies discussed throughout the text contain ZPE corrections. Solvent effects including contributions of non-electrostatic terms have been estimated in single point calculations on the gas phase optimized structures, based on the polarizable continuous solvation model PCM using CH_2_Cl_2_ as a solvent [67]. The cavity is created via a series of overlapping spheres.

For the sake of clarity we did not change the functional for the solvent calculations, despite knowing that the dispersion interactions can occur [68–73]. However, here we consider that the qualitative comparison between the set of studied catalysts and even the quantitative trends (side/bottom) should not be affected by this omission.

## Results and Discussion

In this section we first discuss the structure and energetics of the key steps of metallacycle formation, starting with dissociation of the leaving L ligand, pyridine in this manuscript, from precatalysts **1**–**13**, and moving to coordination of the C=C double bond of ethene in systems **1–13**, or of the C=C bond tethered to the Ru atom in systems **14–19**. Then, we will discuss structure and energetics of the four-center transition state for metallacycle formation, and finally structure and energetics of the metallacycle. In all cases, we assumed the naked 14e species as zero of energy.

**Structure of the naked 14e species.** According to all the calculations reported so far, the naked 14e species is very unstable. The dissociation of PR_3_ or pyridine ligands is highly endothermic, approximately 15–20 kcal/mol even if solvent effects with implicit methods are considered. An unfavorable entropic term of roughly 8–10 kcal/mol would reduce this internal binding energy to free energies of binding around 5–12 kcal/mol [13].

According to simple Boltzmann statistics, these energetics implies that the fraction of the naked 14e species in solution at 25 °C should be in the range of 10^−11^ to 10^−13^ of the total precatalyst. Even considering an overestimation of PR_3_ or pyridine binding by roughly 5 kcal/mol, an error that would be quite large for this kind of calculations, still the fraction of the active species should be in the range of 10^−6^ to 10^−11^. Considering that the precatalyst concentration is usually in the order of 10^−1^ to 10^−3^ M, this means that the concentration of the real 14e catalyst should be roughly in the range of 10^−7^ to 10^−13^. These numbers suggest that it is very unlikely that the naked 14e species is the “real” active species, and that probably some other species is indeed coordinated to the Ru atom in place of the pyridine or phosphine to first dissociate.

**Pyridine and phosphine binding.** The binding energy of the first (trans to the PR_3_ or NHC ligand) and of the second (trans to the ylidene group) pyridine ligands to the naked 14e species of **1**–**19** are reported in the 2nd and 3rd column of Table 1. These values indicate that the first pyridine is bonded quite strongly to the Ru atom. Indeed, it is competitive with PCy_3_ binding in the prototype 1st and 2nd generation systems based on **1** and **7** [74–76]. In precatalysts **1–7** the binding energy of the first pyridine is roughly 20 kcal/mol, with a small effect of the ligand bulkiness, which only changes for system **8** bearing more sterically demanding isopropyl groups, displaying a value of 15.0 kcal/mol. The pseudo-halide systems, instead, show a remarkably different behavior. The pyridine is quite weakly bound to **9**, *E*_1_ = 14.3 kcal/mol, and this binding energy decreases to 7.7 kcal/mol only in **10**. Differently, the pyridine is bound very strongly to the Ru atom, by more than 30 kcal/mol, in systems **11** and **12**. Of course, the pyridine is coordinated trans to the NHC ligand in **9** and **10**, whereas it is cis coordinated in **11** and **12**. Considering the pseudo-halide family, our results are in qualitative agreement with the experimental finding of Fogg and co-workers that systems **11** and **12** have to be thermally activated [19], while system **10** is even more active than the prototype 2nd generation system **7**. Finally, the *C*_1_-symmetric system **13** shows *E*_1_ values which are substantially independent from the specific geometry; i.e., whether the methylidene group is on the side of the mesityl or of the methyl group of the NHC ligand, species, **13a** and **13b**, respectively.

**Table 1:** Binding energy, in kcal/mol, of the first, *E*_1_, and of the second, *E*_2_, pyridine/PMe_3_ molecule to the naked 14e species **1**–**13**.

system	pyridine			PMe_3_		
	*E*_1_	*E*_2_	*E*_1_ *+ E*_2_	*E*_1_	*E*_2_	*E*_1_ *+ E*_2_

**1**	20.3	2.5	22.8	27.3	5.1	32.4
**2**	18.3	9.6	27.9	26.8	9.1	35.9
**3**	21.0	7.7	30.7	25.5	13.2	38.7
**4**	19.9	0.2	20.1	29.3	−5.4	23.9
**5**	21.7	−15.4	6.3	32.8	—	—
**6**	20.7	7.5	28.2	28.5	−4.4	26.1
**7**	19.7	6.8	26.5	27.7	3.6	24.1
**8**	15.0	−6.0	21.0	23.6	−4.6	19.0
**9**	14.3	0.6	14.9	17.9	3.6	21.5
**10**	7.7	−5.0	2.7	11.9	—	—
**11**	33.2	−2.9	30.3	41.1	—	—
**12**	35.6	—	—	45.7	—	—
**13a**	21.1	5.8	26.9	28.9	5.0	33.9
**13b**	13.5	6.1	19.6	17.9	4.9	22.8
**14**	8.7	7.3	16.0	17.9	−2.8	15.1
**15**	11.1	12.6	23.7	19.5	11.9	31.4
**16**	11.5	7.8	19.3	20.8	—	—
**17a**	10.4	5.3	15.7	18.5	4.1	22.6
**17b**	10.3	8.6	18.9	19.2	0.0	19.2
**18**	2.2	5.4	7.6	5.1	9.5	14.6
**19**	13.0	−1.5	11.5	19.8	—	—

Table 1 also includes corresponding values for the phosphine dissociation, PMe_3_ for this analysis. The trend is the same as the one explained above for the pyridine dissociation. But quantitatively the phosphine dissociation is more expensive.

The double coordination of pyridine or trimethylphosphine is not possible for all systems. Systems **5**, **10**, **11**, **12**, **16**, and **19** do not accept the second phosphine group because of steric hindrances. We must point out that for system **12** when bonding one Py or PMe_3_ moiety the octahedral environment around the metal is already obtained. Furthermore there are some other systems that exhibit a negative value for *E*_2_, i.e., no stability for the octahedral structure. This instability is related to the elongation of the Ru–P bond distance cis to the NHC ligand. Indeed in all systems the Ru–P bond is much shorter for the phosphine placed trans to the NHC (around 2.45 Å), compared with the Ru–P bond distance cis to the NHC (around 2.60 Å). However for some systems this distance is too long to be more stable than the monophosphine complex. Thus, when this bond distance is over 2.65 Å the bisphosphine precatalyst structure is not stable.

**Substrate coordination.** The coordination energy of the C=C double bond of the substrate to the Ru atom of the various 14e species are reported in the 3rd column of Table 2, while in the 4th column the coordination energy of the cis isomer relative to the trans isomer is reported. We focus first on C_2_H_4_ coordination to the symmetric precatalysts **1–8**. For these systems we considered both trans and cis coordination of C_2_H_4_ to the PR_3_ or NHC ligand, denoted as “T” and “C” in Table 2. In agreement with experimental findings on related systems, we found that cis coordination of the substrate is preferred. This preference is influenced by the bulkiness of the NHC ligand, and is smaller for larger N-substituents. Indeed, the calculated energy difference between the cis and trans geometries in the coordination intermediates of **2–5**, Δ*E* of 4th column in Table 2, follows a clear trend with the bulkiness of the N-substituents, F < Me < CF_3_ < *t*-Bu, Δ*E* = −8.0 < −5.2 < −3.4 < −2.1 kcal/mol, respectively.

**Table 2:** Energies (*E)* in kcal/mol, of the coordination intermediates, transition states and metallacycles with respect to the 14e species and the uncoordinated C=C double bond. For each system, the energies of both isomers with the C=C trans or cis to the NHC ligand are reported. For each species, Δ*E* is the energy difference between the cis and the trans isomer, labeled as C and T, respectively.

system	geometry	**I**		**TS**		**II**	
		*E*	Δ*E*	*E*	Δ*E*	*E*	Δ*E*

**1** P	T	−10.0	0	−4.2	0	−19.6	0
	C	−11.3	−1.3	−6.6	−2.4	−19.8	-0.2
**2** Me	T	−13.4	0	−9.5	0	−25.7	0
	C	−18.6	−5.2	−10.2	−0.7	−23.4	2.3
**3** F	T	−6.6	0	−5.7	0	−17.8	0
	C	−14.6	−8.0	−10.4	−4.7	−23.2	−5.4
**4** CF_3_	T	−11.9	0	−10.9	0	−23.6	0
	C	−15.3	−3.4	−8.7	2.2	−22.5	1.1
**5** *t*-Bu	T	−17.0	0	−13.1	0	−30.2	0
	C	−19.1	−2.1	−5.6	7.5	−18.0	12.2
**6** IMes	T	−15.5	0	−11.4	0	−25.2	0
	C	−13.6	1.9	−7.8	3.6	−20.8	4.4
**7** SIMes	T	−11.8	0	−10.1	0	−25.0	0
	C	−14.7	−2.9	−6.2	3.9	−19.0	6.0
**8** SIPr	T	−9.9	0	−7.2	0	−21.9	0
	C	−14.7	−4.8	−6.9	0.3	−19.5	2.4
**9** FgF5	T	−7.8	0	−6.7	0	−14.2	0
	C	−16.3	−8.5	−9.7	−3.0	−20.2	−6.0
**1**0 FgBr	T	1.1	0	0.6	0	−9.1	0
	C1	−9.7	−10.8	−0.9	−1.5	−11.4	−2.3
	C2	−5.7	−6.8	0.6	0.0	−15.0	−5.9
**11** FgO	T	3.1	0	3.2	0	−20.6	0
	C	−33.5	−36.6	−24.0	−27.1	−39.1	−18.5
**12** FgS	C (S_1_)	−27.5	0	−21.9	0	−35.6	0
	C(S_2_)	−34.3	−6.8	−26.6	−4.7	−37.6	−2.0
	C(O)	−29.4	−1.9	−18.7	−2.8	−37.5	−1.9
**13a** C1	T	−14.6	0	−13.0	0	−26.3	0
	C	−19.7	−5.1	−9.5	3.5	−23.9	2.4
**13b** C1	T	−8.8	0	−4.9	0	−22.4	0
	C	−12.7	−3.9	−4.8	0.1	−20.1	2.3
**14** PR_3_	T	4.5	0	19.0	0	7.7	0
	C-re	9.7	5.2	20.2	1.2	15.9	8.2
	C-si	14.4	9.9	20.7	1.7	17.1	9.4
**15** Me	T	0.1	0	1.0	0	−3.3	0
	C-re	−5.9	−6.0	4.0	3.0	−1.8	2.5
	C-si	−8.1	−8.2	4.5	3.5	2.2	5.5
**16** Mes	T	−1.8	0	0.6	0	−2.7	0
	C-re	−1.5	0.3	12.6	12.0	8.4	11.1
	C-si	2.4	4.2	19.9	19.3	12.1	14.8
**17a** C1	T	0.0	0	2.7	0	−0.2	0
	C-re	−4.6	−4.6	11.2	8.5	7.4	7.6
	C-si	−0.8	−0.8	12.8	10.1	5.5	5.7
**17b** C1	T	−0.1	0	0.5	0	−4.7	0
	C-re	−2.1	−2.0	2.4	1.9	−3.8	0.9
	C-si	−2.4	−2.3	6.8	6.3	7.4	12.1
**18** Me1	T	1.3	0	2.1	0	−2.7	0
	C-re	−2.7	−4.0	6.9	4.8	0.9	3.6
	C-si	−7.7	−9.0	13.2	11.1	7.3	10.0
**19** Pr1	T	−0.7	0	0.7	0	−4.2	0
	C-re	−0.9	−0.2	14.7	14.0	11.8	16.0
	C-si	−2.8	−2.1	16.7	16.0	12.9	17.1

Steric effects have little influence on the absolute C_2_H_4_ binding energy, *E* of 3rd column in Table 2, since rather similar values are calculated for **2** and **5** (−18.6 and −19.1 kcal/mol, respectively). Differently, electronic effects influence the ability of the Ru atom to capture C_2_H_4_, particularly in the trans isomer. For example, the electron withdrawing F substituents in **3** results in a trans C_2_H_4_ coordination energy of only 6.6 kcal/mol, whereas in the sterically similar system **2** the trans C_2_H_4_ coordination energy is 13.4 kcal/mol. The highest coordination energies are calculated for **2** and **5**, which present electron donating substituents, whereas systems **6** and **7**, with aromatic N-substituents, lie in between. This suggests that electron donating N-substituents increase electron density at the carbene C atom of the NHC, which results in a higher electron density on the σ contribution of the HOMO of the NHC. When 1st and 2nd generation catalysts are compared, system **1**, with the PCy_3_ ligand, behaves rather similary to the 2nd generation catalyst **7**, with a SIMes NHC ligand.

**Figure 1:**
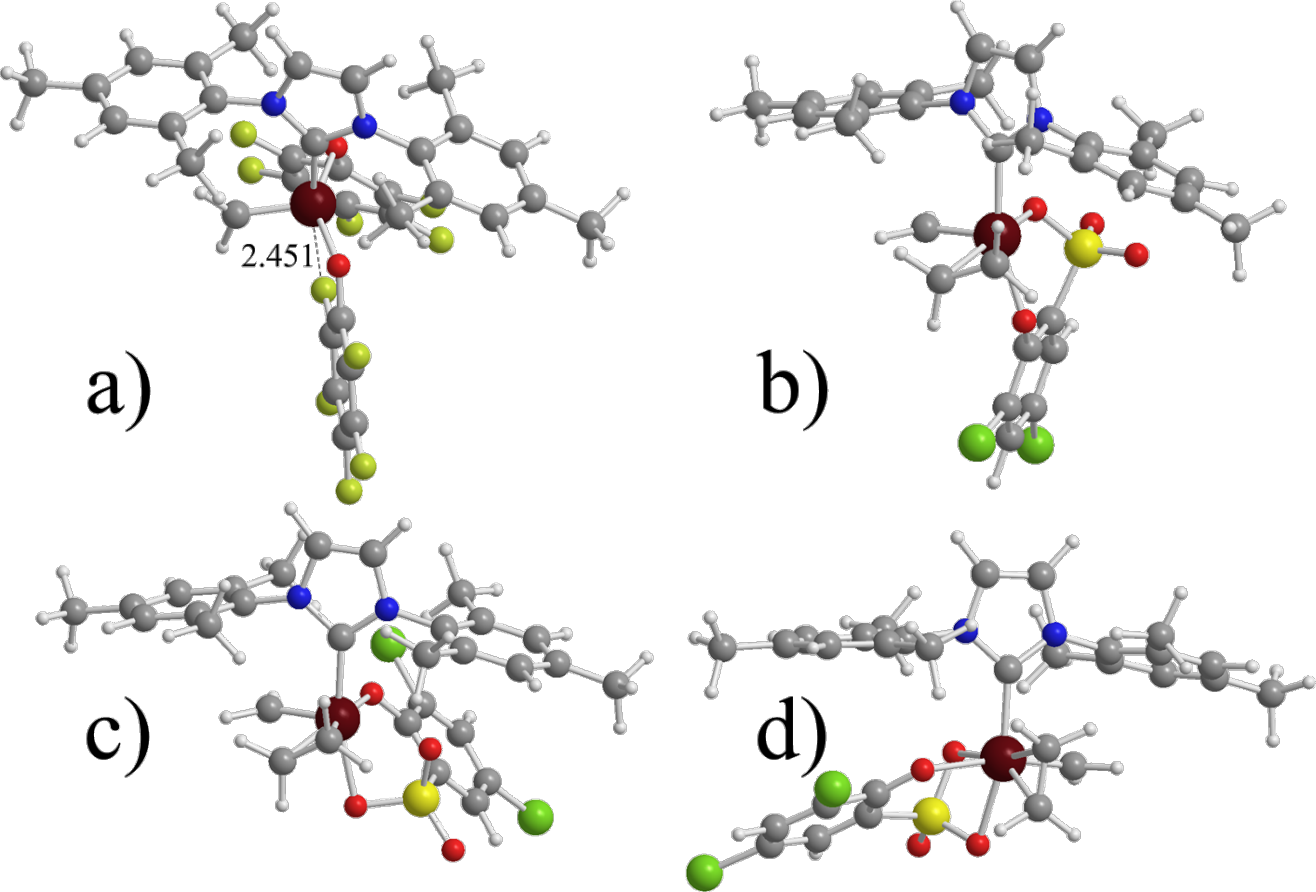
a) Naked 14e species for system **9** (distance in Å). b) trans (T); c) cis(S) (C(S)); and d) cis(O) (C(O)) C_2_H_4_ coordinated species for **12**.

Substitution of the Cl ligands of **7** with the far bulkier O(C_6_F_5_) ligands, such as in **9**, increases the preference for cis C_2_H_4_ coordination from 2.9 to 8.5 kcal/mol, while the absolute C_2_H_4_ binding energy in the cis isomer increases from 14.7 to 16.3 kcal/mol. Interestingly, in the naked 14e species one of the F atoms of the O(C_6_F_5_) ligands is engaged in a Ru^…^F interaction trans to the NHC, which makes the bottom pathway for the entering olefin more difficult, see Figure 1. This kind of Ru^…^F interactions was first reported by Grubbs and co-workers [77–78]. Finally, replacing just one Cl atom of **7** with a C_6_Br_5_ ring, such as in **9**, results again in a preferred cis coordination, and the most stable isomer presents the Cl atom cis to the SIMes ligand.

Moving to the pseudo-halide systems with a chelating ligand, the most striking difference is in the absolute C_2_H_4_ coordination energy, roughly 30 kcal/mol, which is about 15 kcal/mol better than in the non-chelating ligands. The chelating ligand has a minor effect on the cis/trans preference for **12**, whereas for **11** trans coordination of C_2_H_4_ is not favored. This is easily explained by considering that the 5-membered ring formed through chelation of the cresol group to Ru makes a trans O–Ru–O disposition geometrically difficult, whereas the 6-membered ring formed by chelation of the sulfoxide ligand in **12** allows for a trans O–Ru–O geometry with little steric strain. **12** presents three cis isomers, denoted as C(S_1_), C(S_2_), and C(O) in Table 2, which correspond to have the phenolic or the sulfonic O atom trans to the NHC ligand (see Figure 1). Our calculations indicate a preference for the C(S_2_) isomer, which is 4.9 and 6.8 kcal/mol more stable than the C(O) and the C(S_1_) isomers, respectively. The preference for the C(S_2_) isomer can be explained by the weaker donicity of the sulphonic O atom with respect to the phenolic O atom, which results in a softer ligand trans to the SIMes ligand.

**Figure 2:**
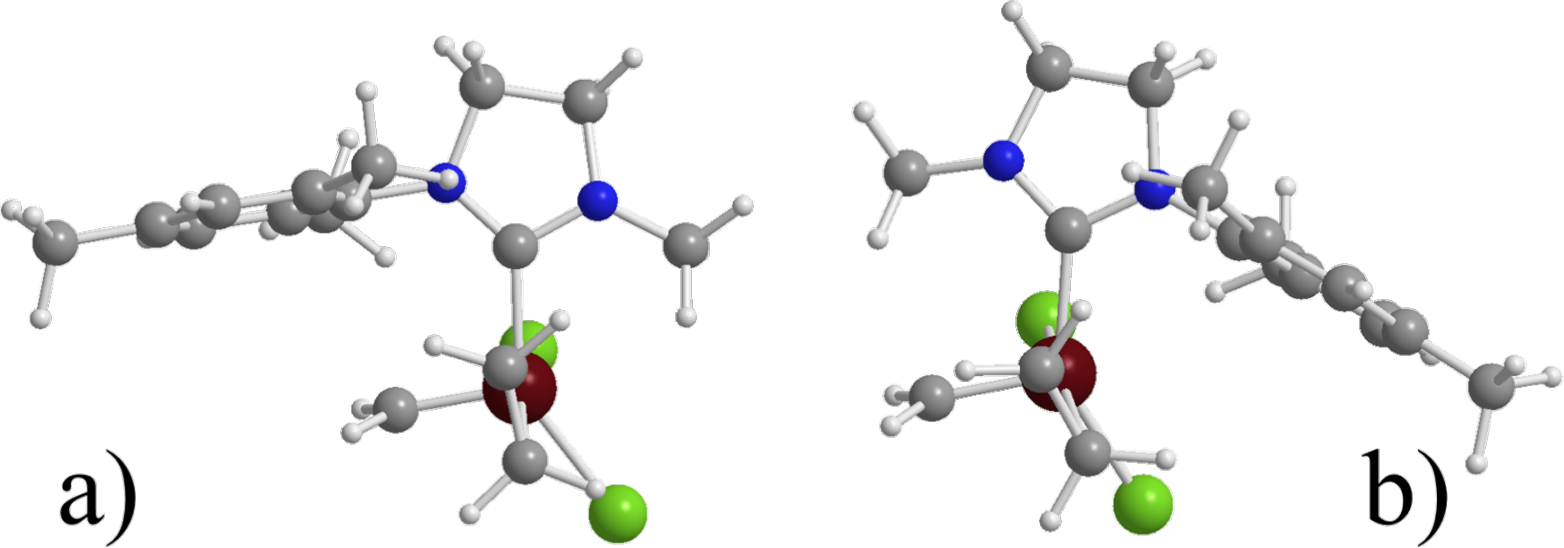
Coordinated species for species a) **13a** and b) **13b**.

Focusing on system **13**, bearing a *C*_1_-symmetric ligand, we found that the cis coordination still is favored, and the most stable structure corresponds to **13a**, which presents the methylidene moiety on the side of the mesityl N-substituent, while isomer **13b**, which presents the methylidene moiety on the side of the Me N-substituent is 7.0 kcal/mol higher in energy. The structure of the two cis coordination intermediates clearly indicates that the C_2_H_4_ molecule nicely avoids steric repulsion with the NHC ligand in **13a**, whereas it experiences repulsive interaction with the methyl on the NHC ligand in **13b** (see Figure 2).

**Figure 3:**
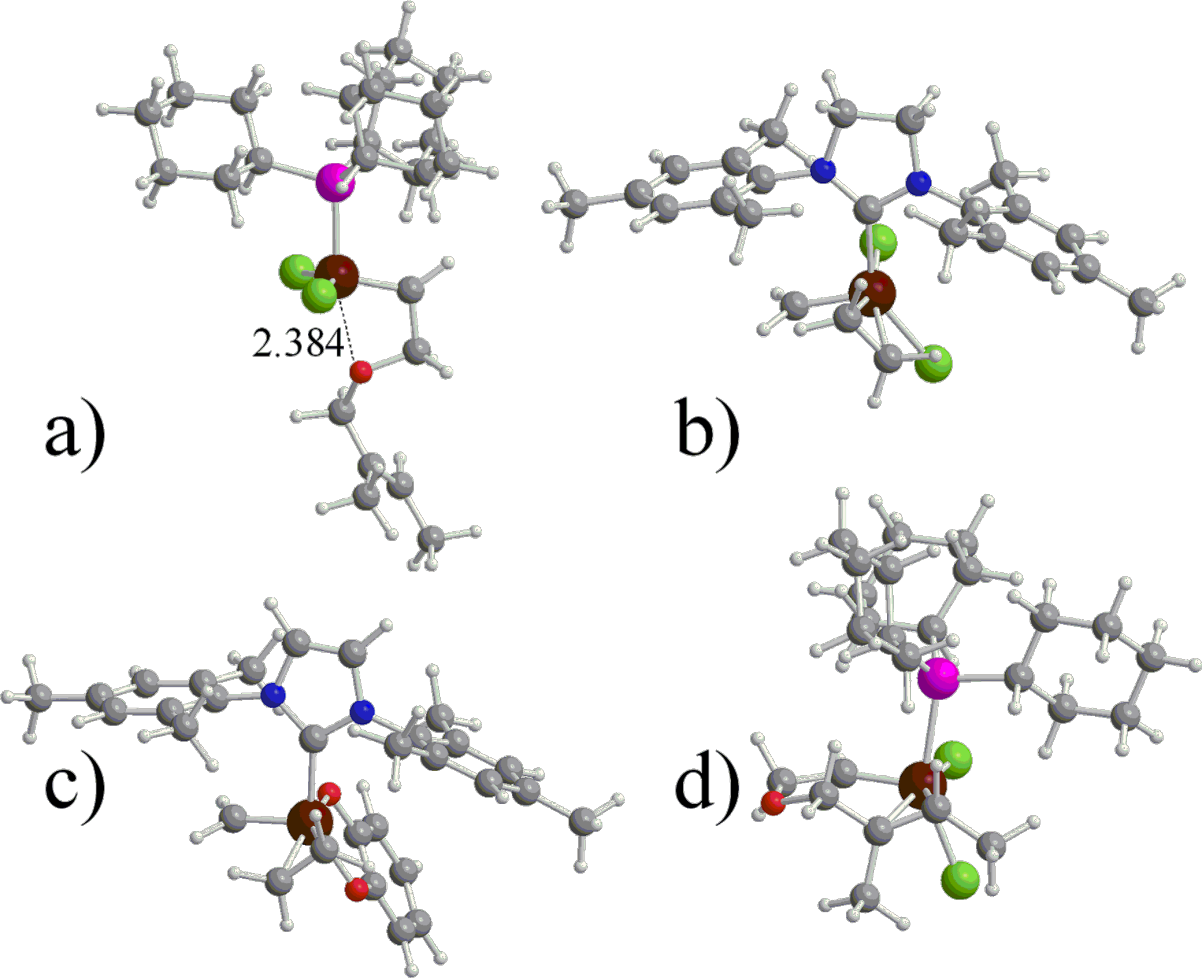
Naked 14e species for system **14** with the O atom of the substrate coordinated to the Ru center (distance in Å), part a; and representative coordination geometries for systems **7**, **11** and **14** with a cis coordinated C_2_H_4_ molecule, parts b–d.

Moving to substrates bulkier than C_2_H_4_, systems **14**–**19**, the cis geometry is favored only in the systems that present small methyl N-substituents, i.e., **15**, **17a** and **18**. This indicates that bulkier substrates can be accommodated in the cis position with difficulty. Furthermore, in almost all the cases coordination of the C=C double bond, either in the cis or trans position, is disfavored (positive *E* values in the 3rd column of Table 2). This striking difference relative to C_2_H_4_ coordination, which is always favored (negative *E* values in the 3rd column of Table 2), is due to the coordination of the O atom of the substrate in **14**–**19**. In other words, with a heteroatom containing substrate the heteroatom can coordinate to the Ru center, as in the Hoveyda-type precatalysts [6], maybe stabilizing the active species, see Figure 3a. In any case, coordination of the C=C double bond requires displacement of the coordinated O atom, and it is likely that the terminal C=C double bond of complex substrates will be dangling, which is in agreement with the NMR experiments of Piers and co-workers on strictly related systems [13]. Focusing on a selection between the two prochiral faces of the C=C bond in **14**–**19**, we always found that coordination takes places in the face that presents the two methyl groups pointing away from the NHC ligand. Of course, this results in reduced steric interactions with the NHC ligand.

In the trans geometries, instead, the C=C double bond of C_2_H_4_ is nearly perpendicular to the Ru–methylidene bond, whereas in the bigger substrate the tether forces the coordinated C=C bond to be almost aligned with the Ru–alkylidene bond. However, the molecular dynamics simulations (vide infra) clearly indicate that in the trans geometries the C_2_H_4_ molecule can freely rotate around the coordination axis to the Ru center. Finally, the Ru–C bond distances are roughly the same (2.20–2.25 Å) in the case of C_2_H_4_ coordination, whereas in the case of the bigger substrate the coordination of the olefin is highly asymmetric, with the internal C roughly 2.50–2.60 Å away from the Ru atom.

**Figure 4:**
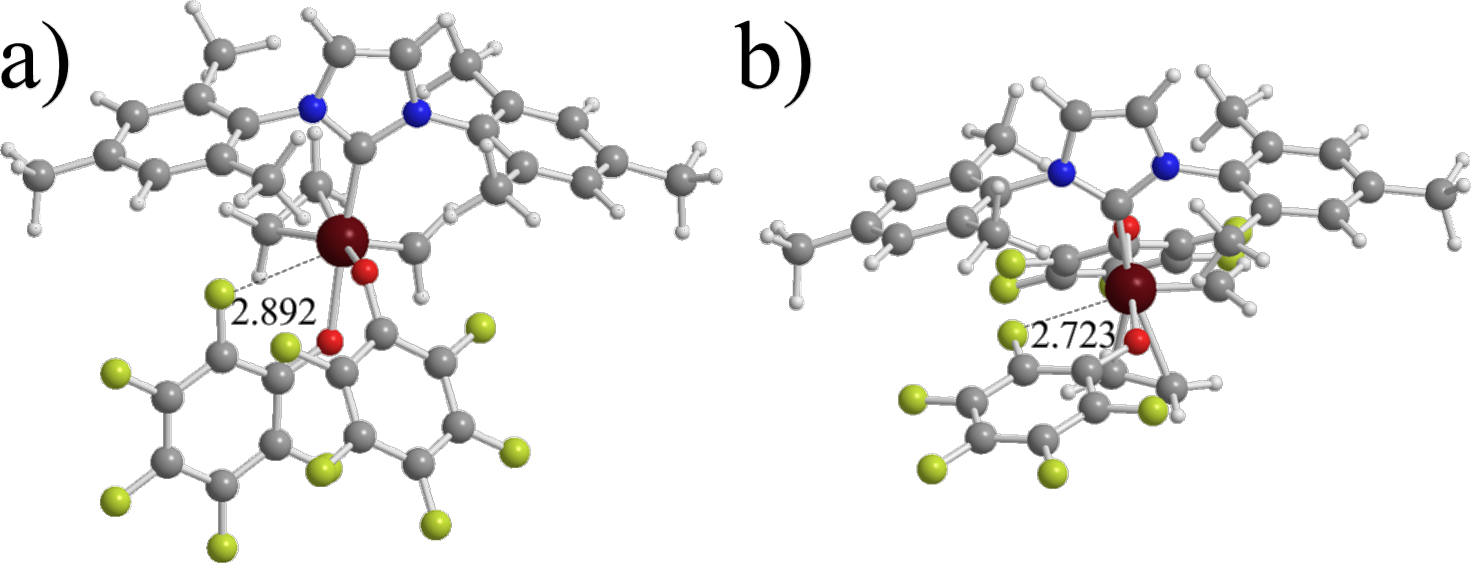
System **9** with a Ru^…^F interaction in the cis and trans geometries, parts a and b, respectively (distance in Å).

**Transition state for metallacycle formation.** The energy of the various transition states for metallacycle formation with respect to the uncoordinated C=C double bond are reported in the 5th column of Table 2, while in the 6th column it is reported the energy of the cis transition states with respect to the trans transition state. The numbers reported clearly indicate that at the transition state the bulkiness of the N-substituents plays a more remarkable role. Indeed, while cis C_2_H_4_ coordination is favored for all the systems we considered, the cis transition state is favored only for some systems. Specifically, for the 1st generation system **1**, for symmetric NHC systems with small N-substituents, such as **3**, for all the pseudo-halide systems, and for system **13**, with a *C*_1_-symmetric NHC ligand. Differently, for non pseudo-halide NHC systems with N-substituents bulkier than Me, such as **4**, **5**, **6**, and **7**, the trans transition state is clearly favored. The increased role of steric effects can be clearly understood if we consider that at the transition state the C=C double bond must be placed almost parallel to the Ru=CH_2_ bond, which results in increased steric repulsion with the NHC ligand, whereas in the coordination intermediates the C=C double bond is almost perpendicular to the Ru=CH_2_ bond, to occupy the free space above the NHC ring, between the N-substituents. The pseudo-halide systems, where the cis coordination is strongly favored, prefer the cis geometry also in the transition state. Finally, for system **9** the Ru^…^F interaction is preserved, see Figure 4. A similar interaction is also retained in the trans transition state, see again Figure 4.

In the case of systems **14–19**, with the bulkier substrate, the trans transition state is clearly favored for all the systems. Also for those that present rather stable cis geometry at the level of the coordination intermediate, such as system **15**. As already indicated, this is due to the steric pressure of the PCy_3_ or NHC ligands on the bulkier substrate. Interestingly, in all the systems considered the energy barrier for metallacyle formation, that is the energy difference between the transition states and the coordination intermediates, is quite small, always below 10 kcal except for system **14**, and usually below 5 kcal/mol. This indicates that all these systems should be highly active, which unfortunately is not the case. This fact suggests that the origin of the remarkably different catalytic activity shown by these systems lays somewhere else.

**Metallacycle.** The energy of the various metallacycles with respect to the uncoordinated C=C double bond are reported in the 5th column of Table 2, while in the 6th column the energy of the cis metallacycle with respect to the trans metallacycle is reported. Beside a few cases, the general trend is that ongoing from the coordination intermediates, to the transition states and finally to the metallacycles, there is an energetic shift towards the trans geometry. In fact, besides the pseudo-halide systems, which strongly favor the cis isomer, the cis metallacycle is favored only for system **3**, with the small F substituents. The stability of the metallacycles relative to the coordination intermediates is strongly influenced by the nature of the substrate. In fact, with C_2_H_4_ as the substrate, systems **1**–**13**, the metallacycle is roughly 10 kcal/mol lower in energy with respect to the coordination intermediates, which suggests that the resting state is the Ru–metallacycle species. This is in agreement with the NMR experiments of Piers and co-workers that found the Ru–metallacycle as the most abundant species in the ethene exchange metathesis promoted by system **7** [32,79]. Differently, with the bigger substrate the metallacycle is of comparable stability or slightly higher in energy with respect to the coordination intermediate, also in agreement with the data of Piers and coworkers [33].

**Figure 5:**
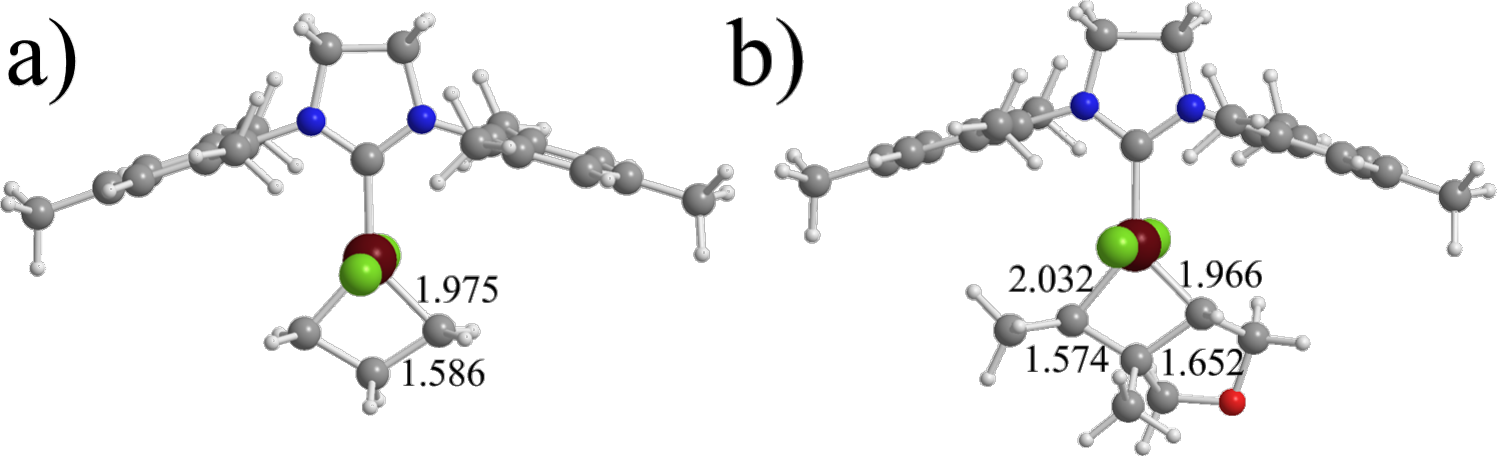
Representative geometries of the metallacycles **7** and **15**, parts a and b, respectively (distance in Å).

Structurally, the Ru–C(metallacycle) bonds in **1**, **2**, **3** and **7** are rather similar, around 1.97–1.98 Å, see Figure 5a, while in **5** they are remarkably longer with 2.01 Å, probably due to the steric pressure of the *t*-Bu N-substituents. In all the cases the C–C bonds of the Ru–cyclobutane are around 1.58–1.59 Å. Differently, in the presence of the bigger substrate the metallacycle is quite asymmetric, see Figure 5b. The C–C length of the former C=C double bond is quite shorter, around 1.57 Å in **14** and **16**, than the C–C bond just formed, which is around 1.65–1.66 Å. This finding is in good agreement with the NMR experiments by Piers and co-workers [33].

**Figure 6:**
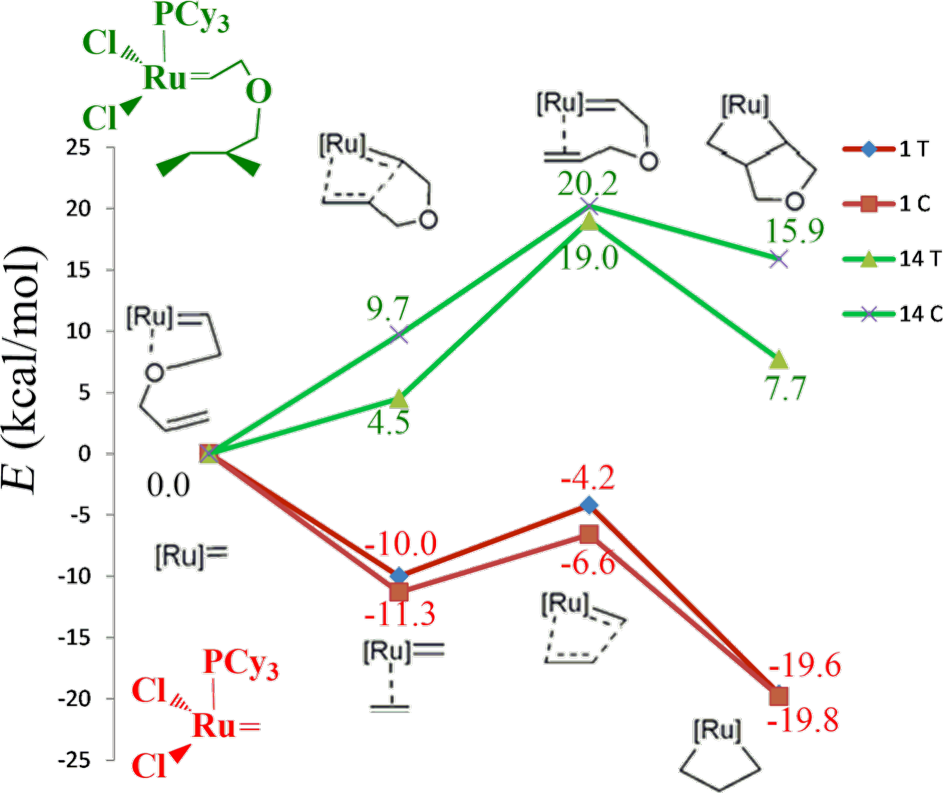
Energy profiles for systems **1** and **14**.

**Figure 7:**
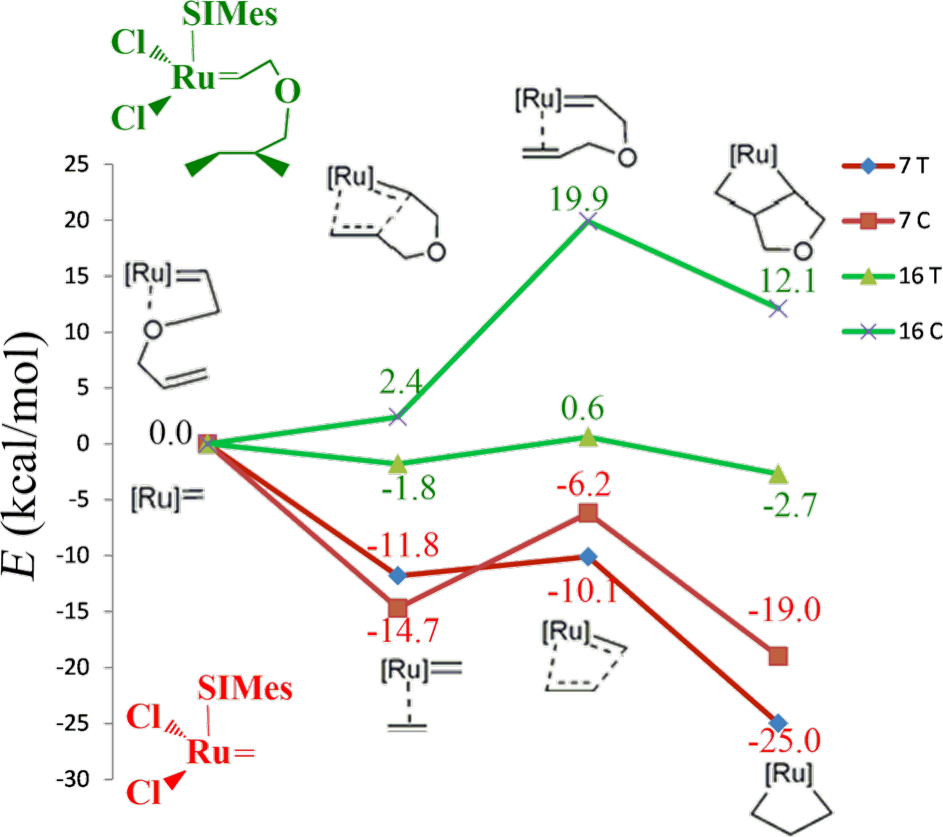
Energy profiles for systems **7** and **16**.

After this reporting on the energetic and structure of each intermediate separately, we combine this information into the energy profile that connects the naked 14e precatalysts and the uncoordinated substrate with the metallacycles. For reasons of simplicity, we selected the most representative cases, which we believe are the 1st and 2nd generation systems with PCy_3_ and SIMes as ligands, both for the small substrate C_2_H_4_, systems **1** and **7**, and the bigger substrate, systems **14** and **16**. In the case of C_2_H_4_ as substrate, coordination of the olefin is clearly favored both for the 1st and 2nd generation systems **1** and **7**, see Figure 6 and Figure 7, although it is clear that the 2nd generation catalysts coordinate to the substrate somewhat better. With the more complex substrate C=C binding has to compete with the O binding, which substantially results in no energy gain for the C=C coordination. Moving to the transition states for metallacycle formation requires climbing the rather low energy barrier of 4.7 and 1.7 kcal/mol for **1** and **7**, respectively, and in both cases the transition state is lower in energy than the naked 14e precatalyst, by 6.6 and 10.1 kcal/mol, respectively. This indicates that metallacycle formation is favored with respect to C_2_H_4_ dissociation. Finally, both transition states collapse into very stable metallacycles. However, beside an overall similarity there is the sharp difference that in **1** the cis path is favored, whereas in **7** the trans path is favored. Furthermore, the energy gain associated with metallacycle formation is quite higher in the 2nd generation catalyst rather than for the 1st generation catalyst. Moving to the same catalysts with the bigger substrate, systems **14** and **16**, metallacycle formation is an overall uphill path. Coordination of the terminal C=C double bond with respect to coordination of the O heteroatom is disfavored by 4.5 kcal/mol in **14** and favored by 1.8 kcal/mol in **16**, respectively. The transition states are 20.2 and 19.9 kcal/mol higher in energy with respect to the 14e species, and the metallacycles are 7.7 kcal/mol above and 12.1 kcal/mol above the 14e species.

**Figure 8:**
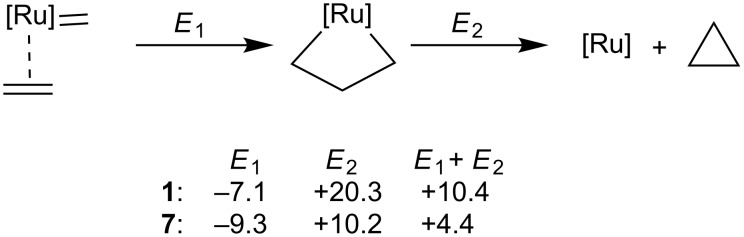
Metallocycle and cyclopropane formation energy profile (energies in kcal/mol).

To better understand the different stability of the metallacycle relative to the coordination intermediate of the 1st and 2nd generation catalysts we investigated the thermodynamics of the reaction shown in Figure 8. *E*_1_ is the energy gain associated to metallacycle formation, already discussed, while *E*_2_ is the energy loss due to the hypothetical release of cyclopropane from the Ru–metallacycle species. The larger *E*_1_ and *E*_2_ values for the 2nd generation system **7** clearly indicate that the Ru–C σ-bonds are stronger in the presence of an NHC ligand. The origin of this difference is in the better ability of the NHC ligand to donate electron density to the Ru center, which formally is in the high formal oxidation state of +4 in the metallacycle.

## Conclusion

After discussing a wide range of representative systems of second generation Grubbs catalysts the conclusion is that any compound sterically demanding either through the alkylidene group or the olefin or even the NHC ligand means a stabilization of the bottom-bound isomer with respect to the side-bound. However, with the simplest olefin and alkylidene groups this less favored isomer plays a key role, especially if taking into account the solvent effect. Therefore, calculations indicate that the preferred reaction pathway is an equilibrium described by steric, electronic, and solvent effects. In spite of the expectation that the hydrogen atom is the less sterically demanding it can produce H–H repulsion with the alkylidene group, which the mesityl or even the methyl groups do not. And comparing these last two complexes the possible H–H repulsions are possible when there is a methyl group in the NHC ligand, but never in the case of mesityl groups. Therefore, for the latter substituent, any H–H repulsion is avoided, and there are favorable C–H interactions even though the mesityl group is sterically more demanding. When including *t*-Bu in the NHC ligand the effect of a high sterically demanding group is present, and for this compound no side-bound isomers occurs.

The differentiation between the power of the three effects, steric, electronic and solvent, has turned out be a hazard to make predictions difficult. However the CF_3_ groups have become key to explain the electronic effects, as well as the *t*-Bu for steric effects. Steric effects owing to interaction between bulky NHC ligands [16] and bulky substrates make the bottom reaction pathway more likely surpassing the other effects. Finally the solvent effect helps the stabilization of the side-bound isomer, but at a higher degree for the high sterically demanding complexes. As already stated by Goddard, polar solvents favor the side reaction pathway or at least reduce the electronic preference for the bottom reaction pathway [13]. Therefore, bearing less sterically demanding substrates and/or ligands, the side reaction pathway, as suggested by Grubbs and co-workers [12,14], might be competitive.

Even though over the last three decades thousands of papers have presented and described the olefin metathesis catalysis, neither a general catalyst for any metathesis reaction has been found [80–82], nor are perfect rules available to predict the behavior of a given catalyst [83–92], although great efforts in characterizing the decomposition reactions [93–97], to validate computational protocols [37,45,68,98–102], and in the experimental synthesis and characterization [33,103] have been taken. Thus, this study provides valuable insight and yields at least a general recipe to obtain a side attack of the olefin towards the NHC ligand.
